# Comparative Short-Term Clinical Outcomes of Hybrid Hyaluronic Acid and Platelet-Rich Plasma Injections in Knee Degenerative Conditions: An Exploratory Real-World Retrospective Study

**DOI:** 10.3390/medicina62071240

**Published:** 2026-06-26

**Authors:** Francesco Librale, Alberta Monaco, Antonio Di Lorenzo, Maurizio Ranieri, Marisa Megna, Riccardo Marvulli, Angelo Paolo Amico

**Affiliations:** 1 Physical Medicine and Rehabilitation Residency Program, University of Bari Aldo Moro, 70124 Bari, Italy; 2 Department of Interdisciplinary Medicine, University of Bari Aldo Moro, 70124 Bari, Italy; 3 Azienda Ospedaliero-Universitaria Consorziale Policlinico di Bari, 70124 Bari, Italy; maurizio.ranieri@uniba.it (M.R.); riccardo.marvulli@policlinico.ba.it (R.M.); paolo-amico@alice.it (A.P.A.); 4 Department of Translational Biomedicine and Neuroscience (DiBraiN), University of Bari Aldo Moro, 70124 Bari, Italy

**Keywords:** knee osteoarthritis, hyaluronic acid, platelet-rich plasma, intra-articular injections, viscosupplementation, degenerative knee conditions, patellofemoral pain

## Abstract

*Background and Objectives*: Knee osteoarthritis (KOA) and other degenerative chondropathies are major causes of pain and disability. When core conservative treatments are insufficient, intra-articular hyaluronic acid (HA) and platelet-rich plasma (PRP) are commonly used as adjunctive options, although evidence remains difficult to interpret because of heterogeneity in patients, products, preparation protocols, and treatment schedules. This exploratory retrospective study described short-term clinical outcomes after two standardized intra-articular protocols, hybrid HA and autologous PRP, in a real-world outpatient physiatry setting. *Materials and Methods*: This monocentric retrospective study included 40 treated knees (19 HA, 21 PRP) from 31 unique patients at the Policlinico di Bari between October 2022 and November 2024. The HA group received two injections of a hybrid high-/low-molecular-weight HA formulation, whereas the PRP group received three injections of autologous PRP. Outcomes were pain intensity, assessed by the Numerical Rating Scale (NRS), and function, assessed by the Western Ontario and McMaster Universities Osteoarthritis Index (WOMAC), from baseline to end-of-cycle follow-up. *Results*: Both groups showed short-term clinical improvement. Mean NRS scores decreased from 6.26 to 2.26 in the HA group and from 6.76 to 2.29 in the PRP group, with no significant between-group difference in change from baseline (*p* = 0.509). WOMAC improved by 25.42 ± 20.39 points in the HA group and 20.19 ± 16.18 points in the PRP group (*p* = 0.372). In the main regression analysis, treatment type was not a significant predictor of outcome; unadjusted and age-/sex-adjusted WOMAC sensitivity models suggested a possible HA advantage that was not retained after full adjustment. *Conclusions*: In this small exploratory cohort, both protocols were associated with short-term improvements, without definitive fully adjusted evidence of between-group superiority. These findings should not be interpreted as evidence of equivalence or definitive comparative efficacy.

## 1. Introduction

Knee degenerative conditions encompass knee osteoarthritis (KOA) and a broader spectrum of degenerative chondropathies, including patellofemoral cartilage degeneration related to overload and patellar maltracking. These disorders are highly prevalent and represent a major source of chronic pain, functional limitation, and disability, with substantial socioeconomic impact [1,2]. Current clinical guidelines emphasize core conservative management (patient education, exercise-based rehabilitation, and weight management when appropriate), while pharmacological options are mainly aimed at symptom control [3,4,5,6]. For patellofemoral pain and overload-related presentations, consensus recommendations similarly prioritize exercise-based therapy and adjunctive physical interventions [7]. Radiographs remain first-line imaging for staging degenerative disease severity [8], commonly using the Kellgren–Lawrence grading system [9].

When symptoms persist despite core treatments, intra-articular injections are frequently used as adjunctive options. Hyaluronic acid (HA) viscosupplementation aims to restore synovial viscoelastic properties and may influence joint homeostasis [10]. Platelet-rich plasma (PRP) delivers an autologous concentration of platelets and bioactive mediators potentially involved in pain modulation and tissue homeostasis; PRP efficacy can vary according to preparation characteristics, including leukocyte concentration and processing method [11,12]. Given this heterogeneity, standardized reporting of PRP preparation and composition has been advocated to improve comparability across studies [13]. In real-world practice, treatment selection is often individualized and influenced by clinical presentation, patient preferences, and local protocols. In addition to hyaluronic acid and platelet-rich plasma, other emerging injective options, including collagen-based products and polynucleotides, are also being explored for the management of degenerative knee conditions [14,15].

In our interventional physiatry outpatient setting, a standardized hybrid high-/low-molecular-weight HA formulation is used for heterogeneous degenerative knee presentations, while a standardized PRP protocol is offered as an alternative biologic approach. Although HA and PRP have been widely investigated, the interpretation of comparative evidence remains limited by heterogeneity in patient phenotype, injectable products, preparation protocols, number of injections, and follow-up schedules. Therefore, real-world data from standardized local protocols may provide pragmatic information on short-term clinical outcomes, while remaining exploratory and not definitive. The present monocentric retrospective study aimed to describe short-term changes in pain intensity and function after these two standardized injection protocols in routine outpatient physiatry practice and to exploratorily assess between-protocol differences.

## 2. Materials and Methods

### 2.1. Study Design and Setting

This monocentric exploratory retrospective observational study was conducted at the Azienda Ospedaliero-Universitaria Consorziale Policlinico di Bari (Bari, Italy) using routinely collected clinical data from a local interventional physiatry outpatient database. Consecutive treated knees receiving intra-articular hyaluronic acid (HA) or platelet-rich plasma (PRP) between October 2022 and November 2024 were identified from this routinely maintained database. No formal a priori sample size calculation was performed because this was an exploratory retrospective study based on all consecutive eligible treated knees available in the local clinical database during the predefined study period. Therefore, the sample size was determined by the number of eligible cases with complete baseline and end-of-cycle follow-up data. Data were extracted from routine clinical records and de-identified at the time of extraction for research purposes; the resulting dataset was analyzed retrospectively.

### 2.2. Participants

The study included heterogeneous degenerative knee presentations, including symptomatic KOA, patellofemoral overload/maltracking-related chondropathy, and mixed degenerative presentations, as documented in routine clinical records. Consecutive treated knees from adult outpatients were eligible if they met all of the following criteria: (i) clinical and/or imaging diagnosis of symptomatic degenerative knee chondropathy; (ii) age 18–70 years; (iii) chronic knee pain (>2 months) associated with functional limitation and indication for an intra-articular injection; (iv) insufficient benefit from prior non-injectable conservative treatments (e.g., systemic pharmacological therapy, physiotherapy, lifestyle modification); (v) no evidence of advanced disease with clear indication for major surgery (e.g., arthroplasty); (vi) treatment performed according to one of the predefined protocols (two HA injections or three PRP injections); and (vii) availability of complete baseline and end-of-cycle follow-up data for pain and function outcomes. When patients contributed two treated-knee observations, both were included in the primary treated-knee-level analysis. The potential non-independence of repeated patient-level observations was addressed through sensitivity analysis, as described in the Statistical Analysis Section.

Exclusion criteria were inflammatory rheumatologic disease or systemic arthropathy; advanced degenerative disease with surgical indication; intra-articular injections in the previous 6 months (including corticosteroids, HA, or PRP); use of injectables other than those specified in the protocol; recent knee fracture or surgery (<6 months); prior partial or total knee arthroplasty of the index knee; history of septic arthritis; contraindications to intra-articular injections (e.g., unmodifiable coagulopathy/anticoagulation, local skin infection, known allergy to materials); pregnancy or breastfeeding; severe uncontrolled systemic illness; and incomplete treatment cycle or missing follow-up data.

### 2.3. Interventions

For HA, only knees treated with the standardized hybrid HA protocol were included to reduce heterogeneity. The product was a sodium hyaluronate (hyaluronic acid sodium salt) medical device containing a total of 64 mg hyaluronic acid (32 mg high–molecular-weight HA + 32 mg low–molecular-weight HA; 3.2% *w*/*v*, i.e., 32 mg/mL) in 2 mL of buffered physiological solution (sodium chloride, sodium phosphate, and water for injections). The HA components were fermentation-derived and non-chemically modified. The HA cycle consisted of two intra-articular injections administered approximately two weeks apart, performed under a standardized aseptic technique. When clinically indicated, joint effusion was aspirated before injection as part of routine practice.

For PRP, a standardized three-injection protocol was used, also spaced approximately two weeks apart. PRP was prepared according to an internal protocol: 180 mL of autologous venous blood was collected and processed at the hospital transfusion service using a dedicated cell separator, the Angel Concentrated Platelet Rich Plasma (cPRP) System (Arthrex, Inc., Naples, FL, USA). The resulting product was classified as leukocyte-poor PRP (LP-PRP) according to the transfusion service preparation protocol; quantitative post-processing leukocyte counts were not available for this retrospective analysis. Three PRP aliquots (approximately 5 mL each) were obtained, with an estimated platelet concentration approximately 18-fold higher than baseline values according to the separator protocol. Ultrasound assessment of the suprapatellar recess was routinely performed to guide access and document effusion. Residual aliquots were stored at −40 °C and thawed within two hours prior to injection. All injections were performed by the same operator using a standardized aseptic technique.

During both treatments, patients were advised to avoid intense physical activity and to abstain from nonsteroidal anti-inflammatory drugs (NSAIDs); paracetamol (up to three doses/day) was allowed as rescue analgesia.

### 2.4. Outcomes

Primary outcomes were pain intensity, assessed by the 0–10 Numerical Rating Scale (NRS) [16], and function, assessed by the Western Ontario and McMaster Universities Osteoarthritis Index (WOMAC) total score [17]. For HA, assessments were performed at t0 (before the first injection), t1 (two weeks later, at the second injection), and t2 (two months after the second injection; endpoint). For PRP, assessments were performed at t0 (first injection), t1 (two weeks later, second injection), t2 (two weeks later, third injection), and t3 (two months after the third injection; endpoint). For between-group comparisons, the endpoint was defined as the change from baseline to the end-of-cycle follow-up (t2 for HA; t3 for PRP).

### 2.5. Statistical Analysis

Data were organized in a dedicated electronic database using Microsoft Excel (Microsoft Corporation, Redmond, WA, USA; version not available; https://www.microsoft.com/microsoft-365/excel; accessed on 24 June 2026), analyzed using Stata/MP 17.0 (StataCorp LLC, College Station, TX, USA), and graphically displayed using RStudio 2026.01.0 (Posit Software, PBC, Boston, MA, USA). Continuous variables are reported as mean ± SD and categorical variables as counts and percentages. The primary endpoints were the absolute changes from baseline to the end-of-cycle follow-up (ΔNRS and ΔWOMAC), defined as t2 for HA and t3 for PRP. After assessment of normality, between-group comparisons of mean changes were performed using independent-samples *t* tests; *p* < 0.05 was considered statistically significant.

For each outcome (ΔNRS and ΔWOMAC), three hierarchical ordinary least squares (OLS) linear regression models were fitted with treatment group (PRP vs. HA) as the main predictor: Model 1 (unadjusted), Model 2 (adjusted for age and sex), and Model 3 (further adjusted for predefined clinical covariates available in the retrospective database and considered potentially relevant to baseline clinical profile or knee symptom burden, including BMI, smoking status, symptom duration in months, diabetes, hypertension, intense physical activity, prior physiotherapy, and prior NSAID use). Given the limited sample size, adjusted models were interpreted as exploratory.

To address the potential non-independence of repeated patient-level observations, a sensitivity analysis restricted to one treated-knee observation per patient was performed by randomly excluding one observation from patients contributing two treated-knee observations. This sensitivity dataset included 31 observations. The same hierarchical regression approach was then repeated in the sensitivity dataset.

Intense physical activity was operationally defined as ≥2 h/week performed at >60% of age- and sex-predicted maximal heart rate. Results are reported as beta coefficients with 95% CI and *p* values.

### 2.6. Ethics

The study was conducted in accordance with the Declaration of Helsinki and applicable data protection regulations. This retrospective observational study was based on routinely collected clinical data and did not involve any additional diagnostic or therapeutic procedures beyond standard clinical care. The study documentation, including the letter of intent and manuscript, was submitted to the Comitato Etico Territoriale Regione Puglia, Azienda Ospedaliero-Universitaria Consorziale Policlinico di Bari. In its meeting held on 22 April 2026, the Ethics Committee formally took note of the submitted documentation and assigned study number 7952. Data were analyzed in de-identified form. Written informed consent for the intra-articular procedures was obtained as part of routine clinical care.

## 3. Results

### 3.1. Baseline Characteristics

Baseline characteristics are summarized in Table 1. The cohort included 40 treated knees from 31 unique patients, of whom 9 contributed two treated-knee observations. At treatment-group level, the HA group included 19 treated knees from 15 unique patients, whereas the PRP group included 21 treated knees from 16 unique patients. The mean age was 56.40 ± 10.31 years, with patients in the HA group being older on average than those in the PRP group. Sex distribution also differed between groups, with a higher proportion of females in the HA group. The frequency of high-intensity physical activity was higher in the PRP group. Diagnostic phenotype distribution was also uneven between groups, with symptomatic KOA more frequent in the HA group and mixed degenerative presentations more frequent in the PRP group. The remaining baseline variables, including BMI, symptom duration, comorbidities, smoking status, prior physiotherapy, and NSAID use, were broadly similar between groups. Beyond the predefined 2-month end-of-cycle follow-up, knee arthroplasty was recorded in 2/19 treated knees in the HA group and 1/21 in the PRP group. These events are reported descriptively only and were not included in the statistical analyses.

### 3.2. Clinical Outcomes

**Figure 1:**
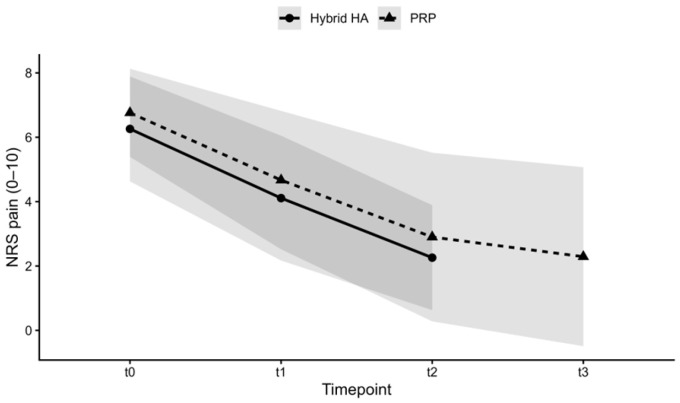
NRS pain score over time in the hybrid HA and PRP groups (mean ± SD). The solid line with circles represents the hybrid HA group, whereas the dashed line with triangles represents the PRP group. Grey shaded areas indicate ± SD around the mean values for each group.

**Figure 2:**
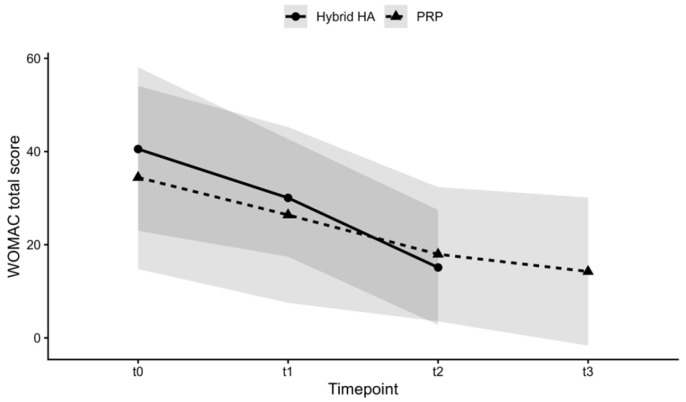
WOMAC total score over time in the hybrid HA and PRP groups (mean ± SD). The solid line with circles represents the hybrid HA group, whereas the dashed line with triangles represents the PRP group. Grey shaded areas indicate ± SD around the mean values for each group.

Mean NRS and WOMAC values at each assessment time point are reported in Table 2, and their time courses are shown in Figure 1 and Figure 2, respectively. Changes from baseline to the end-of-cycle endpoint are summarized in Table 3. Mean NRS reduction was 4.00 ± 1.94 in the HA group and 4.48 ± 2.50 in the PRP group (*p* = 0.509). Mean WOMAC reduction was 25.42 ± 20.39 for HA and 20.19 ± 16.18 for PRP (*p* = 0.372).

### 3.3. Multivariable Analysis

**Figure 3:**
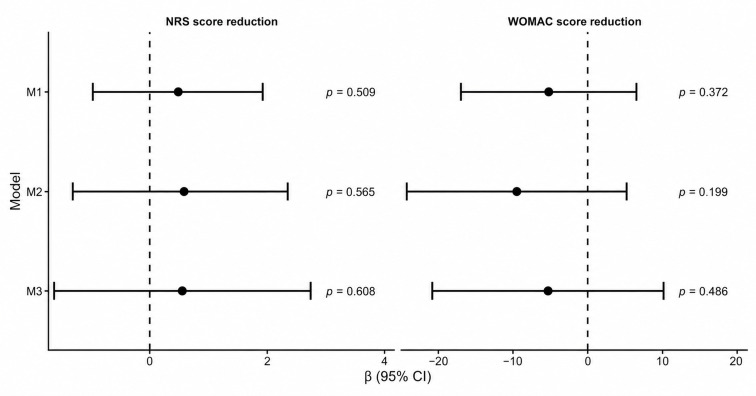
Main-analysis linear regression estimates for treatment group (PRP vs. HA) on changes from baseline to the end-of-cycle follow-up in pain (ΔNRS) and function (ΔWOMAC). Points indicate β coefficients and horizontal lines indicate 95% confidence intervals. The dashed vertical line indicates no between-group difference.

For each outcome (ΔNRS and ΔWOMAC), three hierarchical OLS regression models were fitted: an unadjusted model, a model adjusted for age and sex, and a fully adjusted exploratory model including the covariates described in the Statistical Analysis section. Between-group effect estimates from the unadjusted regression model were small and not statistically significant: for ΔNRS, β = 0.48 (95% CI −0.97 to 1.92); for ΔWOMAC, β = −5.23 (95% CI −16.96 to 6.50). Across all main-analysis models, the estimated effect of PRP relative to HA was not statistically significant. In the sensitivity analysis restricted to one treated-knee observation per patient, no statistically significant between-group difference was observed for ΔNRS. For ΔWOMAC, the unadjusted and age-/sex-adjusted sensitivity models suggested a greater improvement in the HA group compared with PRP; however, this association was attenuated and was no longer statistically significant in the fully adjusted model including baseline clinical covariates. Regression results, including sensitivity analyses, are summarized in Table 4. Regression coefficients from the main analysis are shown in Figure 3.

## 4. Discussion

In this exploratory real-world retrospective cohort, both a standardized hybrid high-/low-molecular-weight HA protocol and an autologous PRP protocol were associated with short-term improvements in pain and function in patients with symptomatic degenerative knee conditions. In the main analysis, no statistically significant between-group differences were observed for changes in NRS or WOMAC, and the estimated effect of PRP relative to HA was not statistically significant across unadjusted, partially adjusted, and fully adjusted models. In the sensitivity analysis restricted to one treated-knee observation per patient, no significant between-group difference was observed for pain reduction. For functional improvement, unadjusted and age-/sex-adjusted sensitivity models suggested a possible advantage of HA over PRP, but this finding was not retained after further adjustment for baseline clinical covariates and should be interpreted cautiously given the small sample size, baseline imbalance, and exploratory nature of the analysis. Importantly, the absence of statistically significant between-group differences in the fully adjusted models should not be interpreted as evidence of equivalence or non-inferiority between HA and PRP, as the study was not designed or powered for equivalence testing. Overall, these findings provide pragmatic short-term real-world data on two standardized injection protocols, while remaining exploratory rather than confirmatory.

These findings should be interpreted in the context of an expanding but still heterogeneous comparative literature. Several systematic reviews and meta-analyses have suggested a relative advantage of PRP over HA for pain and functional outcomes, particularly at medium-term follow-up, although the magnitude and consistency of this effect vary across studies [18,19,20,21]. At the same time, not all comparative studies have demonstrated a clear superiority of PRP. In particular, randomized studies by Filardo et al. and the longer-term follow-up by Di Martino et al. reported significant clinical improvement in both groups without stable or clinically decisive between-group differences, supporting the view that intra-articular treatment itself may provide relevant symptomatic benefit even when one injective option does not clearly outperform the other [22,23]. Our findings are broadly aligned with studies reporting clinical improvement in both treatment groups without clear short-term superiority of one injective approach over the other. However, because of the retrospective design, limited sample size, and absence of an untreated or alternative-treatment comparator group, they should not be interpreted as definitive evidence supporting the comparative effectiveness of either protocol.

A plausible explanation for these mixed findings across studies lies in the marked methodological heterogeneity of the available literature. PRP protocols differ substantially in platelet concentration, leukocyte content, preparation method, number of injections, and treatment intervals, while HA products may vary in molecular weight, formulation, and viscoelastic properties [13,24]. Such variability limits direct comparability across studies and may partly explain why some reports favor PRP whereas others show broadly similar outcomes between treatments. In this respect, one strength of the present study is the use of consistent local protocols for both interventions, reducing within-group procedural heterogeneity. However, our short-term follow-up does not allow us to determine whether differences in durability might emerge over longer time horizons, as suggested by some previous studies [18,19,20].

Another relevant methodological aspect concerns the different treatment schedules used in the two groups. The HA protocol consisted of two injections, whereas the PRP protocol consisted of three injections, both administered approximately two weeks apart. Therefore, the present study compares two routinely used standardized clinical protocols, rather than two injectable products delivered under matched treatment schedules. This difference may influence treatment burden, timing of clinical assessment, patient expectations, and temporal response patterns, and should be considered when interpreting the observed short-term outcomes.

From a clinical and rehabilitation-oriented perspective, our findings may be particularly relevant because they derive from routine outpatient physiatry practice rather than from a highly selected experimental population. In everyday care, treatment selection is often influenced not only by efficacy expectations, but also by patient profile, treatment availability, local expertise, cost considerations, and shared decision-making. In this context, observing short-term improvement in both groups, with no statistically significant between-group differences in the main analysis, may still provide pragmatic information for routine care, provided that these findings are interpreted cautiously and not as evidence of equivalence. Short-term symptom reduction should not be viewed as an endpoint in itself, but rather as a potential therapeutic window that may facilitate adherence to core conservative management, especially exercise-based rehabilitation, which remains central in current guideline-based care [3,4,5,6,7,25].

Another clinically relevant aspect of our study is the heterogeneity of the treated population, which included both older individuals with KOA and younger patients with patellofemoral overload or patellar maltracking. This case mix may be viewed from two complementary perspectives. On the one hand, it limits phenotype-specific inference and precludes conclusions about whether one treatment might be preferable in specific degenerative subgroups. On the other hand, it reflects the spectrum of patients commonly encountered in outpatient interventional physiatry and therefore enhances the pragmatic relevance of the findings. In this sense, the findings may have pragmatic relevance for outpatient interventional physiatry, but they should not be generalized to specific diagnostic phenotypes without confirmation in larger, prospectively defined and more homogeneous cohorts. Future prospective studies with larger samples, longer follow-up, and phenotype-based stratification are warranted to better define which patients may benefit most from each approach [26].

### Limitations

This study has limitations inherent to its retrospective observational design, including the absence of randomization, the risk of selection bias, and the potential for residual confounding despite adjustment. Treatment allocation was based on routine clinical practice and may have been influenced by clinical presentation, patient preferences, treatment availability, cost considerations, and shared decision-making. Therefore, confounding by indication cannot be excluded.

No formal a priori sample size calculation or power analysis was performed because this was an exploratory retrospective study based on all consecutive eligible treated knees available in the local clinical database during the predefined study period. Consequently, the small sample size may have limited the statistical power to detect modest between-group differences, increasing the risk of type II error. For this reason, the absence of statistically significant between-group differences should not be interpreted as evidence of equivalence or absence of clinically meaningful differences.

The unit of analysis was the treated knee joint. Because some patients contributed two treated-knee observations, within-patient correlation could not be fully excluded. To address this issue, a sensitivity analysis restricted to one treated-knee observation per patient was performed. This analysis did not materially change the interpretation of pain outcomes. For functional outcomes, unadjusted and age-/sex-adjusted sensitivity models suggested a possible between-group difference favoring HA, but this finding was not retained in the fully adjusted model and should be interpreted cautiously in view of the limited sample size and baseline imbalance.

Given the limited sample size and the number of covariates included in the fully adjusted models, multivariable estimates should be interpreted as exploratory and may be affected by model instability or overfitting. The adjusted analyses were intended to assess whether the treatment-group estimate remained broadly consistent after accounting for selected baseline differences, rather than to provide definitive causal inference.

The clinical spectrum of the sample was heterogeneous, including symptomatic KOA, patellofemoral overload/maltracking-related chondropathy, and mixed degenerative presentations. Although broad diagnostic phenotypes were reconstructed from routine clinical records and reported descriptively, the retrospective database did not consistently include standardized radiographic severity grading, including Kellgren–Lawrence classification, or sufficiently granular imaging-based characterization. Therefore, reliable phenotype-stratified or radiographic severity-stratified analyses could not be performed.

Because this was a retrospective real-world cohort comparing two routinely used injection protocols, untreated, corticosteroid-injection, or rehabilitation-only comparator groups were not available. Accordingly, the observed within-group improvements should be interpreted as clinical trajectories after treatment rather than as isolated estimates of treatment-specific effects. The study design does not allow the specific contribution of HA or PRP to be fully separated from contextual or non-specific effects, regression to the mean, natural symptom fluctuation, or possible concurrent conservative measures. In addition, the two protocols differed in treatment schedule, with two injections in the HA group and three injections in the PRP group, limiting direct comparison of the injectable products themselves.

Follow-up was limited to a short-term endpoint, namely two months after treatment completion, and imaging-based structural outcomes were not assessed. Although the PRP protocol was standardized and the product was classified as LP-PRP, quantitative post-processing cellular composition data, including leukocyte and erythrocyte counts, were not available, precluding a more granular characterization of the injective product.

Accordingly, the present results should be interpreted as exploratory real-world evidence and should not be generalized to longer-term comparative effectiveness, treatment equivalence, or phenotype-specific response without further confirmation. Future prospective controlled studies with larger cohorts, longer follow-up, standardized phenotype classification, and appropriate sample size planning are warranted.

## 5. Conclusions

In this small exploratory real-world retrospective cohort, both the standardized hybrid high-/low-molecular-weight HA protocol and the autologous PRP protocol were associated with short-term improvements in pain and function in patients with symptomatic degenerative knee conditions. In the main analysis, no statistically significant difference between PRP and HA was observed for either pain reduction or functional improvement. In the sensitivity analysis restricted to one treated-knee observation per patient, no significant between-group difference was observed for pain reduction. For functional improvement, unadjusted and age-/sex-adjusted sensitivity models suggested a possible advantage of HA over PRP; however, this finding was not retained after further adjustment for baseline clinical covariates. Therefore, these results should not be interpreted as evidence of equivalence, non-inferiority, or definitive comparative efficacy between HA and PRP. Larger prospective controlled studies with standardized phenotype classification, appropriate sample size planning, and longer follow-up are needed to clarify the comparative role and durability of response of these injective protocols within multimodal conservative management.

## Figures and Tables

**Table 1:** Baseline characteristics and diagnostic phenotypes of treated knees. Data are reported at the treated-knee level (HA, *n* = 19; PRP, *n* = 21; overall, *n* = 40).

Variable	HA	PRP	Overall
Age, years (mean ± SD)	61.68 ± 6.47	51.62 ± 10.92	56.40 ± 10.31
Female sex, n (%)	13 (68.4%)	5 (23.8%)	18 (45.0%)
Male sex, n (%)	6 (31.6%)	16 (76.2%)	22 (55.0%)
BMI, kg/m^2^ (mean ± SD)	27.95 ± 5.08	25.63 ± 4.33	26.73 ± 4.78
Symptom duration, months (mean ± SD)	23.37 ± 24.31	28.67 ± 21.75	26.15 ± 22.86
**Diagnostic phenotype, n (%)**			
Patellofemoral chondropathy/overload-maltracking	8 (42.1%)	12 (57.1%)	20 (50.0%)
Symptomatic KOA	10 (52.6%)	1 (4.8%)	11 (27.5%)
Mixed degenerative presentation	1 (5.3%)	8 (38.1%)	9 (22.5%)
**Comorbidities and previous treatments, n (%)**			
Diabetes mellitus, n (%)	5 (26.3%)	2 (9.5%)	7 (17.5%)
Hypertension, n (%)	10 (52.6%)	7 (33.3%)	17 (42.5%)
High-intensity physical activity ≥2 h/week, n (%)	5 (26.3%)	13 (61.9%)	18 (45.0%)
Prior physiotherapy, n (%)	6 (31.6%)	6 (28.6%)	12 (30.0%)
Prior NSAID use, n (%)	14 (73.7%)	17 (81.0%)	31 (77.5%)
Smoking (current or former), n (%)	10 (52.6%)	9 (42.9%)	19 (47.5%)

**Table 2:** Mean NRS and WOMAC scores at each assessment time point. Data are reported as mean ± SD. For HA, t0 indicates baseline, t1 the second injection, and t2 the 2-month post-cycle follow-up. For PRP, t0 indicates baseline, t1 the second injection, t2 the third injection, and t3 the 2-month post-cycle follow-up.

Time Point	NRS (HA)	NRS (PRP)	WOMAC (HA)	WOMAC (PRP)
t0	6.26 ± 1.63	6.76 ± 1.37	40.53 ± 17.55	34.43 ± 19.65
t1	4.11 ± 1.94	4.67 ± 2.15	30.05 ± 12.63	26.38 ± 18.86
t2	2.26 ± 1.63	2.90 ± 2.62	15.11 ± 12.32	17.95 ± 14.41
t3	NA	2.29 ± 2.78	NA	14.24 ± 15.90

**Table 3:** Changes from baseline to the end-of-cycle follow-up in pain (ΔNRS) and function (ΔWOMAC). Data are reported as mean ± SD.

Outcome	HA	PRP	*p*-Value
ΔNRS	4.00 ± 1.94	4.48 ± 2.50	0.509
ΔWOMAC	25.42 ± 20.39	20.19 ± 16.18	0.372

**Table 4:** Linear regression models predicting changes from baseline to the end-of-cycle follow-up in pain (ΔNRS) and function (ΔWOMAC), with treatment group as exposure (PRP vs. HA). M1: unadjusted; M2: adjusted for age and sex; M3: additionally adjusted for BMI, smoking status, symptom duration, diabetes, hypertension, intense physical activity, prior physiotherapy, and prior NSAID use. Sensitivity analysis was based on a dataset restricted to one observation per patient (*n* = 31), obtained by randomly excluding one observation from patients contributing two treated-knee observations.

Outcome	Analysis	Model	β	95% CI	*p*-Value
ΔNRS	Main analysis	M1	0.48	−0.97; 1.92	0.509
		M2	0.52	−1.31; 2.35	0.565
		M3	0.55	−1.63; 2.74	0.608
	Sensitivity analysis	M1	−0.32	−1.77; 1.12	0.649
		M2	−0.83	−2.63; 0.97	0.353
		M3	−0.44	−2.60; 1.71	0.672
ΔWOMAC	Main analysis	M1	−5.23	−16.96; 6.50	0.372
		M2	−9.50	−24.21; 5.21	0.199
		M3	−5.33	−20.81; 10.14	0.486
	Sensitivity analysis	M1	−13.30	−25.21; −1.38	0.030
		M2	−17.13	−32.02; −2.24	0.026
		M3	−13.25	−30.62; 4.11	0.127

## Data Availability

The de-identified data presented in this study are available from the corresponding author upon reasonable request. The data are not publicly available due to ethical and privacy restrictions related to the retrospective use of clinical data.

## Abbreviations

The following abbreviations are used in this manuscript:

KOA	Knee Osteoarthritis
HA	Hyaluronic Acid
PRP	Platelet-Rich Plasma
LP-PRP	Leukocyte-Poor Platelet-Rich Plasma
NRS	Numerical Rating Scale
WOMAC	Western Ontario and McMaster Universities Osteoarthritis Index
BMI	Body Mass Index
NSAIDs	Nonsteroidal Anti-Inflammatory Drugs
SD	Standard Deviation
OLS	Ordinary Least Squares
CI	Confidence Interval
APC	Article Processing Charge
NA	Not Available
